# Anthocyanin-Rich Extracts from Bilberries and Blackcurrants in Human Health: A Narrative Review of Their Anti-Inflammatory and Antioxidant Effects

**DOI:** 10.3390/jcm15052083

**Published:** 2026-03-09

**Authors:** Carlos Escobar-Cervantes, Clotilde Vázquez-Martinez, Silvia Gómez-Senent, Alexandra Eva Henriquez-Linares, María Fasero-Laiz

**Affiliations:** 1 Cardiology Department, University Hospital La Paz, 28046 Madrid, Spain; 2 Endocrinology and Nutrition Department, University Hospital Fundación Jiménez Díaz, 28040 Madrid, Spain; clotilde.vazquez@quironsalud.es; 3 Gastroenterology Department, University Hospital La Paz, 28046 Madrid, Spain; silviagsenent@gmail.com; 4 Obstetrics and Gynecology Department, Hospital Quirónsalud Olympia, 28046 Madrid, Spain; henriquezgine@gmail.com; 5 Corofas-Menopause Clinic, 13700 Tomelloso, Spain; info@menopausiacorofas.com; 6 Medicine Department, Francisco de Vitoria University, Majadahonda, Km 1.800, 28223 Madrid, Spain

**Keywords:** aging, anthocyanin, atherosclerotic cardiovascular disease, diabetes, mental health, nonalcoholic fatty liver disease, inflammation, oxidative stress

## Abstract

Inflammation and oxidative stress are key mechanisms in aging, contributing to neurodegenerative diseases, cardiovascular diseases, type 2 diabetes, obesity, and other conditions. In the aging process, the increase in reactive oxygen species and the decrease in antioxidant pathways damage cellular components, accelerating deterioration. Persistent inflammation and oxidative stress also favor the progression of diseases such as atherosclerosis, where LDL oxidation and infiltration in the arteries generate plaques that can lead to myocardial infarction or stroke. In addition, inflammation and oxidative stress can affect the immune system, as well as the development of chronic inflammatory diseases and nonalcoholic fatty liver disease, and may affect mental health, healthy menopause and muscle recovery. Research from both human studies and laboratory tests indicates that taking 80–320 mg per day of anthocyanin-rich extracts from bilberries and blackcurrants (Anthocyanin-EBB) can moderately enhance cholesterol levels, lower markers of inflammation, boost blood vessel health, increase insulin responsiveness, and reduce indicators linked to cardiovascular and metabolic risks. They also have antioxidant, anti-inflammatory and neuroprotective effects, helping in the prevention and management of chronic diseases. As a result, supplementation with anthocyanin-rich extracts may be a promising strategy to promote healthy aging and reduce the risk of development and progression of conditions related to oxidative stress and chronic inflammation. Nevertheless, due to the limited patient populations and short follow-up periods in most existing studies, long-term clinical trials are necessary to determine the definitive advantages of Anthocyanin-EBB in clinical practice.

## 1. Importance of Oxidative Stress and Chronic Inflammation on Aging and Different Clinical Conditions

Chronic inflammation and oxidative stress are interconnected processes that contribute to the pathogenesis and progression of multiple chronic diseases. Understanding their roles is essential to developing effective strategies for their prevention and treatment.

### 1.1. Aging

Aging is a complex process that involves physiological, molecular, and cellular changes over time, and has been associated with an increase in susceptibility to various pathological conditions, including neurodegenerative and cardiovascular diseases (CVD), dyslipidemia, or type 2 diabetes (T2D) [1,2,3]. One of the key mechanisms involved in aging is oxidative stress, which results from the imbalance between the production of reactive oxygen species (ROS) and the body’s ability to detoxify or repair them. Cellular oxidation, caused by the accumulation of ROS and also the alteration of antioxidant pathways, such as the Nrf2 pathway, can damage essential cellular components, including DNA, proteins, and lipids. This damage contributes to the loss of cellular and tissue function, accelerating the aging process. Additionally, mitochondria, the main sources of ROS, become dysfunctional with age, contributing to increased oxidative stress and cellular deterioration. Moreover, the body’s antioxidant capacity decreases with age, further aggravating oxidative damage. Since oxidative damage is a central factor in the accumulation of molecular damage that characterizes aging, strategies to reduce oxidative stress and restore the antioxidant pathways, as well as mitochondrial function could be key in the promotion of healthy longevity [4,5,6,7,8,9,10].

On the other hand, chronic inflammation commonly occurs with aging and contributes to diseases like cardiovascular (CV) and neurodegenerative conditions, T2D, and impaired immunity, thereby speeding up aging. This ongoing, low-level inflammation, marked by increased pro-inflammatory molecules and proteins, is associated with cellular senescence, immune system dysfunction, and reduced inflammatory resolution. Therefore, reducing this proinflammatory state is essential for promoting healthy aging [11,12,13,14].

### 1.2. Prediabetes, Type 2 Diabetes, Overweight, and Obesity

Chronic low-grade inflammation and oxidative stress are also important in the development and progression of different conditions such as prediabetes, T2D, being overweight, and obesity. Thus, in prediabetes and T2D, inflammation contributes to insulin resistance, making it difficult for the body to use glucose efficiently. The oxidation of lipids and proteins also generates cell damage, aggravating metabolic dysfunction [15,16,17]. In overweight people and those with obesity, excessive adipose tissue secretes inflammatory substances such as cytokines, which promote a systemic inflammatory state. This process favors the appearance of insulin resistance and other metabolic complications. In addition, oxidative stress can damage cells and tissues, perpetuating a cycle of inflammation and damage [18,19]. Therefore, interventions that reduce these processes may be beneficial, improving insulin sensitivity and reducing the risk of progression to diabetes [20,21,22].

### 1.3. Atherosclerotic Cardiovascular Disease

Atherosclerotic cardiovascular disease (ASCVD) is the leading cause of death and disability worldwide. The development of cholesterol plaques constitutes the pathophysiologic basis of atherosclerosis. Classically, within the etiopathogenesis of ASCVD, an interrelationship has been established between lifestyle, different risk factors such as hypertension, diabetes, dyslipidemia or smoking, and genetic factors, with significant variations across different populations and socioeconomic groups [23]. However, different studies have shown that oxidative stress and inflammation would also play a determining role in the pathogenesis of ASCVD. Thus, chronic low-grade inflammation in the artery walls promotes the infiltration of immune cells, such as macrophages and lymphocytes, leading to atherosclerotic plaque development. The oxidation of lipoproteins, especially low-density lipoproteins (LDLs), is a key step in this process. Thus, oxidized LDL are more likely to be taken up by macrophages, forming foam cells that constitute the core of atherosclerotic plaques. In addition, oxidized LDLs promote an inflammatory response, stimulating the release of proinflammatory cytokines and enzymes that deteriorate the arterial wall, leading to instability of the atherosclerotic plaque, thus increasing the risk of events such as myocardial infarction or stroke [24,25,26,27,28]. In this context, reducing inflammation and oxidation processes can be an effective therapeutic strategy for preventing the development of CV complications [29,30,31,32].

### 1.4. Immune System

Inflammation and oxidation play critical roles in the normal functioning and regulation of the immune system. Thus, inflammation is an essential biological response in the defense of the body against harmful stimuli, such as infections, damaged cells or injury, and to eliminate pathogens and repair damaged tissues. On the other hand, oxidation, particularly the production of ROS, is a natural part of the immune response, since ROS help destroy pathogens. However, it is necessary to have a proper balance, as excessive oxidation may be harmful, leading to chronic inflammatory conditions. As a result, controlled inflammation and ROS production are essential for an effective immune response. Promoting an adequate balance and proper regulation are crucial to avoiding damage and chronic diseases [33,34,35].

### 1.5. Chronic Inflammatory Diseases

There are some chronic inflammatory diseases, such as rheumatoid arthritis or inflammatory bowel disease, in which inflammation and oxidation play a key role. Thus, these conditions are characterized by persistent inflammation and excessive production of ROS, leading to ongoing tissue damage. The continuous interaction between inflammation and oxidation promotes a vicious cycle that aggravates lesions and delays disease resolution [36]. Fortunately, antioxidants and therapies aimed at reducing oxidation may modulate these processes and may be effective in decreasing inflammation, preventing complications and improving clinical outcomes in these conditions [37,38].

### 1.6. Nonalcoholic Fatty Liver Disease

Nonalcoholic fatty liver disease (NAFLD) is a condition in which fat builds up in the liver in people without excessive alcohol consumption. It is more common in people who are overweight or obese and is the most common form of liver disease in the world [39]. Inflammation and oxidation play a central role in the progression of this condition, especially in its most advanced form, non-alcoholic steatohepatitis. In NAFLD, the accumulation of fat in the hepatocytes causes cellular stress and damage, which activates the inflammatory response. Thus, immune cells, such as macrophages and Kupffer cells in the liver, release cytokines and inflammatory mediators that contribute to chronic inflammation, promoting progression to advanced stages and, eventually, liver fibrosis. Additionally, fat overload in the liver increases the production of ROS, increasing oxidative stress, which impairs lipids, proteins, and DNA in the liver, generating toxic products, aggravating inflammation, promoting cell apoptosis and contributing to disease progression. Therefore, inflammation and oxidation transform a simple accumulation of fat in the liver to an active inflammatory disease that potentially may cause serious complications [40,41,42]. In this context, antioxidants and anti-inflammatories could be considered as potential therapeutic targets to slow the progression of NAFLD to more severe stages [43,44].

### 1.7. Mental Health

Chronic low-grade inflammation has been associated with several neurodegenerative conditions and mood disorders, including cognitive impairment or depression. Therefore, the activation of the inflammatory response within the central nervous system can affect neuronal function, facilitate neurodegeneration, and modify neurotransmitter activity, collectively contributing to cognitive decline and depressive symptoms. In addition, pro-inflammatory cytokines, including IL-6 and TNF-α, can cross the blood–brain barrier and modulate neuronal plasticity and neurogenesis, thereby impacting mental health [45,46]. Excess ROS leads to oxidative stress, damaging lipids, proteins, and DNA in brain cells. This contributes to neurodegeneration, disrupts neuronal communication, impairs neurotransmitter production and is linked to cognitive impairment and disorders like depression [47,48]. Anti-inflammatory and antioxidant therapies are being investigated to enhance cognitive function and mental health [49].

### 1.8. Improvement of Muscle Recovery

Muscle recovery following physical exercise is an essential process for enhancing performance, preventing injuries, and supporting the body’s adaptation to training. Exercise triggers inflammation and oxidation, which aid muscle repair but need regulation to avoid excessive damage and support recovery. Thus, excessive or prolonged inflammation can delay recovery and increase the risk of injury. Furthermore, oxidation via ROS has a dual role: at moderate levels, it aids muscle repair signaling, but excessive ROS causes damage, delaying recovery and increasing fatigue. Recent studies indicate that managing inflammation and oxidation, through nutrition, antioxidants, and active recovery, can enhance muscle recovery [50,51].

### 1.9. Menopause

Estrogens play a pivotal role in maintaining redox homeostasis by enhancing the expression of antioxidant enzymes and modulating mitochondrial reactive oxygen species (ROS) production. Through these mechanisms, they provide natural protection against oxidative stress and lipid peroxidation. The abrupt decline in estrogen levels that occurs during menopause disrupts this protective balance, leading to increased mitochondrial dysfunction, elevated oxidative damage, and excessive generation of free radicals. This rise in oxidative stress represents a fundamental pathophysiological mechanism underlying the onset of metabolic alterations. Consequently, estrogen deficiency in menopause markedly amplifies both oxidative and cardiometabolic risk, contributing to the heightened incidence of cardiovascular disease observed in postmenopausal women [52].

## 2. Anthocyanins: Properties and Characterization of a Standardized Anthocyanin-Rich Extract from Bilberries and Blackcurrants

Anthocyanins constitute a significant class of water-soluble polyphenolic pigments belonging to the flavonoid subclass. These compounds are responsible for conferring colors ranging from orange-red to blue-violet in various plants, including dark-colored fruits and vegetables. The substantial scientific interest in anthocyanins stems from their potent antioxidant and anti-inflammatory activities. The specific preparation under investigation, the anthocyanin-rich extract from bilberries and blackcurrants (Anthocyanin-EBB), is a standardized nutraceutical preparation consisting of purified natural anthocyanins (Medox^®^, MedPalett AS, Norway). This extract is isolated from wild bilberries (*Vaccinium myrtillus*) and blackcurrants (*Ribes nigrum*). The formulation contains a total of 17 different purified anthocyanins. The primary constituents are delphinidin glycosides and cyanidin glycosides, which typically account for approximately 58.0% and 33.0% of the total anthocyanin content, respectively. Several studies have shown that Anthocyanin-EBB has positive effects on inflammation and oxidative stress and has been shown to provide human health benefits, such as protection against chronic diseases [53,54]. In this review we focused on the evidence about Anthocyanin-EBB and its potential for clinical use. For this purpose, a systematic search was conducted on PubMed (MEDLINE), using the MeSH terms [Inflammation] + [Oxidative stress] + [Anthocyanin-EBB] up to December 2025. Original data from clinical trials, observational studies and reviews of interest were reviewed.

## 3. Experimental and Clinical Evidence with Anthocyanin-EBB

Many studies have analyzed the impact of Anthocyanin-EBB on different oxidative and inflammatory parameters, as well as on clinical conditions (Table 1 and Supplementary Table S1) [55,56,57,58,59,60,61,62,63,64,65,66,67,68,69,70,71,72,73,74,75,76,77,78,79,80,81,82,83,84,85,86,87,88,89,90,91,92].

### 3.1. Lipid Profile and Endothelial Function

Several studies have examined how Anthocyanin-EBB affect lipid profiles, endothelium function and inflammatory markers. In a double-blind, randomized clinical trial involving 120 dyslipidemic subjects aged 40–65 years, participants received either 160 mg Anthocyanin-EBB twice daily or a placebo for 12 weeks. The group treated with Anthocyanin-EBB showed a statistically significant increase in high-density lipoprotein (HDL) cholesterol (HDLc) and a significant reduction in LDL cholesterol (LDLc) levels compared to the placebo group. Additionally, cellular cholesterol efflux to serum increased more in the Anthocyanin-EBB group, along with a greater decrease in both the mass and activity of plasma cholesteryl ester transfer protein [55]. In a crossover study, 12 individuals with high cholesterol received 320 mg oral Anthocyanin-EBB or placebo. In this study, the flow-mediated dilation (FMD) increased significantly from 8.3% at baseline to 11.0% after 1 h and 10.1% after 2 h. In a 12-week trial with 150 participants, those given Anthocyanin-EBB showed greater improvements in FMD (28.4% vs. 2.2%), cyclic guanosine monophosphate (cGMP) (12.6% vs. −1.2%), HDLc compared to placebo. Additionally, reductions in soluble vascular adhesion molecule-1 (sVCAM-1) and LDLc were also observed with Anthocyanin-EBB. Another short study observed that activation of the nitric oxide (NO)-cGMP pathway correlated with enhanced endothelium-dependent vasodilation [56]. A randomized, double-blind trial in 150 hypercholesterolemic subjects showed that taking Anthocyanin-EBB (320 mg/d) for 24 weeks significantly reduced high sensitivity C-reactive protein (hsCRP), sVCAM-1, IL-1β, and LDLc, and increased HDLc compared to placebo. The authors also reported additive or synergistic anti-inflammatory effects of Anthocyanin-EBB in vitro [57]. A double-blind, placebo-controlled crossover study of 31 men with prehypertension, but without dyslipidemia at baseline found that four weeks of Anthocyanin-EBB consumption significantly increased HDLc levels [58]. In a double-blind, randomized, placebo-controlled trial involving 122 hypercholesterolemic participants, administration of 160 mg of Anthocyanin-EBB twice daily for 24 weeks resulted in a significant increase in HDLc and a reduction in LDLc compared to placebo. Furthermore, Anthocyanin-EBB supplementation enhanced both HDL-associated paraoxonase 1 (PON1) activity and cholesterol efflux capacity relative to placebo. Notably, HDL-PON1 activity was inversely correlated with lipid hydroperoxide levels and positively correlated with improved cholesterol efflux capacity [59]. In a randomized, double-blind trial involving 58 patients with T2D, participants were administered either 160 mg of Anthocyanin-EBB twice daily or placebo over a 24-week period. Anthocyanin-EBB supplementation resulted in significant reductions in LDLc (7.9%), triglycerides (23.0%), apolipoprotein B-48 (16.5%), and apo C-III (11.0%), alongside an increase in HDLc (19.4%). Additionally, Anthocyanin-EBB intake lowered fasting plasma glucose by 8.5% and the homeostasis model assessment for insulin resistance index (HOMA-IR) by 13%, while elevating serum adiponectin by 23.4% and β-hydroxybutyrate by 42.4%. Furthermore, patients receiving Anthocyanin-EBB also exhibited enhanced antioxidant capacity, and lowered serum markers of oxidative stress, in particular higher total radical-trapping antioxidant parameter and ferric ion reducing antioxidant power values and less serum concentrations of 8-iso-prostaglandin F2α, 13-hydroxyoctadecadienoic acid, and carbonylated proteins [60]. In a 12-week randomized, double-blind trial involving 160 participants with prediabetes or early untreated diabetes, Anthocyanin-EBB (320 mg/day) modestly reduced HbA1c, LDLc, apo A1, and apo B compared to placebo, with greater effects in those with higher metabolic markers [61]. A systematic review of seven studies found that Anthocyanin-EBB supplementation was linked to reduced levels of LDLc and apoB, as well as increased concentrations of HDLc and apoA1 [62]. In a randomized study involving 176 dyslipidemic participants aged 35 to 70 years, individuals were assigned to receive one of three purified Anthocyanin-EBB doses (40 mg/day, 80 mg/day, or 320 mg/day) or a placebo over a 12-week period. The results demonstrated a dose-dependent improvement in HDLc levels and HDL-induced cholesterol efflux capacity [63]. A double-blind, randomized, controlled trial involving 93 individuals with dyslipidemia evaluated the effects of Anthocyanin-EBB supplementation compared to placebo over 12 weeks. The study found that Anthocyanin-EBB reduced collagen-induced platelet aggregation, activated GPIIb/IIIa, ADP-induced platelet aggregation, platelet ROS levels, and mitochondrial membrane potential in a dose-dependent manner [64]. In another study, Anthocyanin-EBB (320 mg/day for 4 weeks) reduced inflammation and improved glucose and lipid metabolism in people with metabolic syndrome by inhibiting NF-κB activation and increasing PPAR-γ gene expression [65]. In a randomized clinical trial involving 169 participants with dyslipidemia, Anthocyanin-EBB supplementation resulted in dose-dependent reductions in plasma ceramides, a novel risk factor for metabolic disorders and CVD, following 12 weeks of treatment. Additionally, notable improvements were observed in plasma lipid profiles, including decreases in non-HDLc, apolipoprotein B, and total cholesterol, as well as enhanced cholesterol efflux capacity [66].

### 3.2. Thrombogenesis and Platelet Function

The supplementation with Anthocyanin-EBB has been associated with notable benefits on thrombogenesis, platelet hyperactivation, and hyper-aggregation. Research indicates that Anthocyanin-EBB intake may be linked to changes in gene expression and biological processes related to cell adhesion, migration, immune response, and cell differentiation, which could positively impact vascular health [67]. Similarly, a randomized, double-blind, placebo-controlled, cross-over trial demonstrated that in a sedentary populations, daily supplementation with 320 mg of dietary Anthocyanin-EBB may reduce biomarkers associated with thrombogenesis, platelet hyperactivation, and hyper-aggregation. The intervention was associated with decreased monocyte-platelet aggregate formation, inhibition of platelet endothelial cell adhesion molecule-1 expression, reduced platelet activation-dependent conformational change and degranulation, as well as lower ADP-induced whole blood platelet aggregation [68]. A randomized, double-blind, placebo-controlled study conducted in an overweight/obese population found that Anthocyanin-EBB supplementation (320 mg/day) for 28 days was associated with inhibition of ADP-induced platelet activation-related conformational change and degranulation by reducing PAC-1 and P-selectin expression. The supplementation also decreased monocyte-platelet aggregate formation and platelet endothelial cell adhesion molecule-1 (PECAM-1) expression. Additionally, Anthocyanin-EBB supplementation reduced platelet aggregation induced by ADP, collagen, and arachidonic acid, suggesting that Anthocyanin-EBB supplementation may lower the risk of thrombosis [69]. In a study specifically performed in patients with metabolic syndrome, treatment with Anthocyanin-EBB supplements for 4 weeks was associated with significant decreases in serum fasting blood glucose, triglycerides, LDLc, and hs-CRP levels, together with a decrease in ADP-induced platelet activation configuration [70]. These effects have also been documented in healthy populations, with a daily supplementation of 320 mg Anthocyanin-EBB over 28 days resulting in significant reductions in platelet activity, platelet aggregation, and mean platelet volume [71]. All these data suggest that Anthocyanin-EBB supplementation exerts anti-atherogenic effects by improving cardiometabolic risk factors, reducing thrombogenicity and improving endothelial function.

### 3.3. Insulin Resistance and Glucose Metabolism

Numerous studies have examined the beneficial impact of Anthocyanin-EBB on insulin resistance, prediabetes, and T2D. Evidence suggests that Anthocyanin-EBB contributes to improved adipocyte function and confers protection against metabolic stress. In vitro research indicates that, while palmitic acid promotes inflammation by activating the NF-κB pathway and triggers insulin resistance through modulation of the phosphatidylinositol 3-kinase-protein kinase B/Akt axis, GLUT-1, and adiponectin mRNA expression, Anthocyanin-EBB can reverse these effects in a dose-dependent manner [72]. In a randomized controlled trial, supplementation with purified Anthocyanin-EBB over 12 weeks resulted in improved serum adiponectin levels and lower fasting glucose in patients with newly diagnosed T2D compared to placebo. This finding is significant, as adiponectin, a protein secreted by adipose tissue, plays a role in enhancing glucose metabolism, reducing blood triglyceride levels, and exhibiting both anti-inflammatory and anti-atherogenic effects, thereby offering protection against cardiovascular diseases [73]. In another study involving 121 participants with elevated fasting glucose, supplementation with Anthocyanin-EBB (320 mg/day) for 12 weeks, compared to placebo, led to improvements in serum insulin-like growth factor binding protein-4 (IGFBP-4) fragments, biomarkers linked to cardiometabolic diseases, as well as reductions in fasting glucose and post-load C-peptide levels [74]. A study of 160 individuals with prediabetes or recently diagnosed diabetes demonstrated that administration of 320 mg Anthocyanin-EBB for 12 weeks, compared to placebo, resulted in a significant increase in serum adipsin and a reduction in visfatin. Furthermore, Anthocyanin-EBB supplementation led to statistically significant improvements in HbA1c (−0.11%), apo A-1 (0.12 g/L), and apo B (−0.07 g/L) levels. These findings are notable as adipsin and visfatin are adipokines secreted by adipose tissue, both of which play crucial roles in metabolic regulation. Specifically, adipsin contributes to the maintenance of beta cell function and may offer protective effects against T2D, whereas visfatin has been linked to insulin resistance and T2D [75].

### 3.4. Inflammation and Oxidative Stress

The vascular benefits and the anti-inflammatory effects of Anthocyanin-EBB have also been analyzed in different studies. Accordingly, Anthocyanin-EBB supplementation has been shown to confer protective effects on endothelial cells by modulating cell signaling pathways in response to mild hyperoxia-induced changes [76]. In a study involving 169 participants with dyslipidemia, 12 weeks of Anthocyanin-EBB supplementation was associated with increases in anti-oxidative and anti-inflammatory capacity. The supplementation showed dose–response relationships with reductions in inflammatory cytokines IL-6, tumor necrosis factor-α (TNF-α), and oxidative stress biomarkers including urine 8-iso-prostaglandin F2α (8-iso-PGF2α), 8-hydroxy-2′-deoxyguanosine (8-OHdG), and serum malonaldehyde (MDA) [77]. Similarly, another study conducted on human diabetic endothelial cells exposed to oxidative and inflammatory stressors demonstrated that Anthocyanin-EBB attenuated oxidative stress and inflammation by inhibiting the NF-κB signaling pathway. This inhibition played a role in reducing the diabetes-induced up-regulation of NF-κB [78]. These dose-dependent antioxidant and anti-inflammatory properties of Anthocyanin-EBB supplementation have also been observed in healthy subjects, suggesting that Anthocyanin-EBB supplementation could be useful in the prevention, or even treatment of chronic inflammatory diseases [79,80]. Additionally, a study using a murine asthma model reported that Anthocyanin-EBB reduced the development of asthma by downregulating T-helper cell type 2 cytokines, proinflammatory cytokines, and cyclooxygenase (COX)-2. These results indicate that Anthocyanin-EBB supplementation may have potential for asthma prevention [81]. Anthocyanin-EBB supplementation has been observed to exhibit anti-inflammatory effects in individuals who are also obese or overweight. As chronic low-grade inflammation is linked to an increased risk of T2D and CVD in this population, targeting inflammation may be considered as a complementary approach to management [82]. Furthermore, Anthocyanin-EBB could reduce proliferation of human colorectal carcinoma cells by promoting ROS accumulation, inducing caspase-3 activation and p21 upregulation [83].

### 3.5. NAFLD and Liver Biomarkers

Anthocyanin-EBB could have favorable effects on NAFLD. Thus, in an experimental study conducted on mice, Anthocyanin-EBB treatment demonstrated protective effects against NAFLD and mitochondrial dysfunction induced by a methionine-choline-deficient diet. The underlying mechanism may involve modulation of the AMP-activated protein kinase (AMPK)/peroxisome proliferator-activated receptor-gamma coactivator-1α (PGC-1α) signaling pathways [84]. A pilot study was conducted to assess the effects of Anthocyanin-EBB supplementation on insulin resistance and liver injury biomarkers in 74 patients with NAFLD. In this double-blind, randomized trial, participants received either Anthocyanin-EBB (320 mg/d) or placebo for 12 weeks. The Anthocyanin-EBB group showed reductions in plasma alanine aminotransferase, cytokeratin-18 M30 fragment, and myeloperoxidase compared to placebo. Additionally, the Anthocyanin-EBB group exhibited a non-significant decrease in fasting blood glucose and HOMA-IR compared to placebo, with a significant difference observed in the oral glucose tolerance test [85]. In another experimental study, Anthocyanin-EBB demonstrated a protective effect against acute acetaminophen-induced hepatotoxicity in rats [86].

### 3.6. Mental Health and Cognition

Different studies have determined the effects of Anthocyanin-EBB on cognitive health. In in vitro studies on neuronal cells, it has been observed that Anthocyanin-EBB may reduce cytotoxicity, restore antioxidant capacity, and improve mitochondrial function, suggesting potential benefits for neurodegenerative diseases, such as Alzheimer disease [87,88]. In an open-label study, 27 individuals with mild cognitive impairment or stable non-obstructive coronary artery disease were treated with 80 mg of natural purified Anthocyanin-EBB, twice daily for 16 weeks. Anthocyanin-EBB were well tolerated, and compliance was high, but the results on memory were inconclusive [89]. The FINGER study was a 24-week, randomized, double-blind, placebo-controlled trial designed to evaluate the effects of purified Anthocyanin-EBB (320 mg/day) on cognitive function in individuals at increased risk for dementia. Anthocyanin-EBB treatment was significantly associated with improvements in LDLc, cardiometabolic score, CRP, IL-6, IL-1b, and Inflam z-score 5. Notably, participants with elevated inflammation markers experienced cognitive benefits from Anthocyanin-EBB supplementation, whereas those with lower inflammation levels did not [90,91]. In a randomized placebo-controlled study involving 206 participants aged 60–80 years with either mild cognitive impairment or two or more cardiometabolic disorders, individuals were assigned to receive four capsules totaling 320 mg/day of naturally purified Anthocyanin-EBB or placebo for 24 weeks. Anthocyanin-EBB supplementation was found to be safe and well tolerated in this group. While no significant difference between groups was observed in episodic memory at the study’s conclusion, there was a statistically significant difference in slopes: the Anthocyanin-EBB group showed improvement, while the placebo group exhibited decline [92].

## 4. Potential Clinical Profiles for the Use of Anthocyanin-EBB

Each capsule of Anthocyanin-EBB contains 80 mg of anthocyanins, standardized from 210 mg of extract. The recommended dosage is typically one to two capsules daily, taken with water. These capsules are vegan, free from gluten and dairy, and made using a patented nitrogen extraction method. It is marketed as a food supplement, not a prescription medicine. Anthocyanin-EBB capsules are usually well-tolerated, with rare side effects such as mild gastrointestinal issues, dark stools, dizziness, or rash reported in studies using 80–320 mg daily. Excessive intake, significantly above the recommended levels, may potentially result in renal complications, alter thyroid hormone regulation, or function as pro-oxidants rather than antioxidants [53,54].

**Figure 1:**
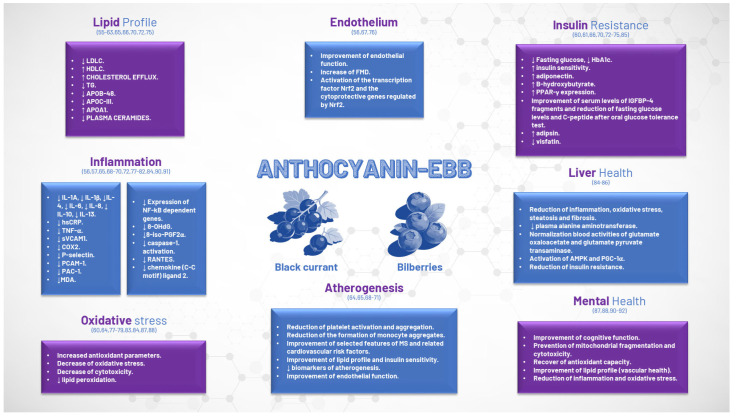
Mechanisms of action of anthocyanin-EBB on different organs and systems. Anthocyanin-EBB: anthocyanin-rich extracts from bilberries and blackcurrants; AMPK: AMP-activated protein kinase; ApoA-1, apolipoprotein A-1; ApoB, apolipoprotein B; FMD: flow-mediated dilation; HDLc: high-density lipoproteins cholesterol; 8-OHdG: 8-hydroxy-2′-deoxyguanosine; hsCRP: high sensitivity C-reactive protein; IGFBP-4: insulin-like growth factor binding protein-4; IL: interleukin; 8-iso-PGF2α: 8-iso-prostaglandin F2α; LDLc: low-density lipoproteins cholesterol; MDA: malondialdehyde; MS: metabolic syndrome; PAC-1: procaspase activating compound-1; PECAM-1: platelet endothelial cell adhesion molecule-1; PGC-1α: peroxisome proliferator-activated receptor-gamma coactivator-1α; PPAR-γ: peroxisome proliferator-activated receptor gamma; RANTES: regulated upon activation, normal T cell expressed and secreted; sVCAM1: soluble vascular cell adhesion molecule-1; TG: triglycerides; TNF-α: tumor necrosis factor-α; T.

**Figure 2:**
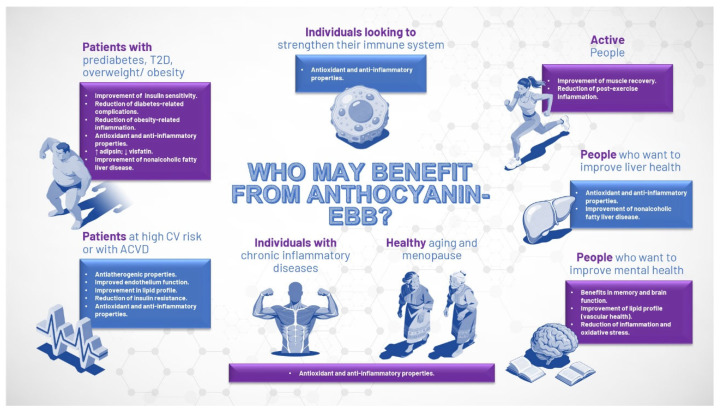
Individuals that may benefit from treatment with anthocyanin-EBB. Anthocyanin-EBB: anthocyanin-rich extract from bilberries and blackcurrants; ACVD: atherosclerotic cardiovascular disease; CV: cardiovascular T2D: type 2 diabetes.

Inflammation and oxidative stress play significant roles in the aging process as well as in the onset and progression of various clinical disorders. Owing to the dose-dependent benefits of Anthocyanin-EBB on a range of biological parameters, including comprehensive lipid profiles, various inflammatory markers, oxidative stress levels, endothelial function, and reductions in biomarkers associated with atherogenesis or liver disease, as well as improved insulin sensitivity (Figure 1) [55,56,57,58,59,60,61,62,63,64,65,66,67,68,69,70,71,72,73,74,75,76,77,78,79,80,81,82,83,84,85,86,87,88,89,90,91,92], the potential advantages for individuals with diverse clinical conditions are considerable (Figure 2). However, it is important to note that while several studies have examined the effects of Anthocyanin-EBB across experimental data and patient populations, these studies generally involved a limited number of participants. Still, the findings have been remarkably consistent, often aligned with the expected mechanism of action and suggesting that Anthocyanin-EBB could be a beneficial supplement for certain patients.

Given their significant anti-inflammatory and antioxidant properties, Anthocyanin-EBB supplementation may offer potential benefits for individuals seeking healthy aging, immune system enhancement, or management of chronic inflammatory conditions [56,57,60,64,68,69,70,72,77,78,79,80,81,82,83,84,87,88,90,91]. Similarly, these properties of Anthocyanin-EBB may support muscle recovery, particularly in individuals who are physically active [93,94]. Insulin resistance plays a key role in the development of T2D, as well as associated complications, especially in predisposed patients, such as those with prediabetes, overweight and obesity. Although lifestyle changes (weight loss and increased physical activity) are essential, they are often insufficient [95,96]. Due to its beneficial effects on insulin sensitivity, and its anti-inflammatory and antioxidant properties, Anthocyanin-EBB supplementation could be an added value in the management of this population [60,61,65,70,72,73,74,75,85].

Despite the advances in the management of patients with ACVD, there is still a residual risk that is not currently covered by current treatments. This is partly because these treatments cannot effectively reduce the pro-inflammatory and prooxidative state that occurs in these patients [97,98]. In this context, Anthocyanin-EBB supplementation could provide an added value by acting specifically on these alterations [64,65,68,69,70,71].

The management of NAFLD is centered on lifestyle modifications, with weight reduction serving as a fundamental strategy. Although no pharmacological therapy specifically targets NAFLD at this time, the comprehensive approach of these patients through the control of all associated CV risk factors seems mandatory [99,100]. Anthocyanin-EBB exhibits several properties, such as reducing inflammation, oxidative stress, steatosis, fibrosis, and insulin resistance, in addition to normalizing transaminase levels, that collectively suggest Anthocyanin-EBB supplementation may represent a promising therapeutic option for this population [84,85,86].

Some evidence indicates that anthocyanins may benefit cognitive function due to their anti-inflammatory, antioxidant effects, and ability to prevent mitochondrial fragmentation and cytotoxicity. Thus, Anthocyanin-EBB supplementation suggests potential benefits in cognitive performance and mood regulation [87,88,90,91,92].

Estrogen deficiency during menopause raises oxidative and cardiometabolic risk, increasing cardiovascular disease in postmenopausal women [52]. Anthocyanin-EBB supplementation may help lower this risk through its antioxidant and anti-inflammatory properties. Research suggests that blackcurrant anthocyanins may act as phytoestrogens by binding to estrogen receptor β (ERβ) and showing weak estrogenic effects. This can help address issues such as bone loss and vascular problems in peri- and early postmenopausal women [101,102]. Additionally, anthocyanin-EBB suggest benefits for cognitive health, potentially addressing difficulties like “mental fog” often experienced during menopause [52].

## 5. Conclusions

Current evidence from clinical and experimental studies supports the use of Anthocyanin-EBB as effective modulators of oxidative stress and inflammation. These extracts have demonstrated significant benefits in improving lipid profiles, endothelial function, insulin sensitivity, and inflammatory markers, with additional neuroprotective and hepatoprotective effects. Their application may be particularly relevant in the context of aging, metabolic syndrome, cardiovascular disease, NAFLD, and cognitive impairment.

While findings are promising, further large-scale, long-term randomized controlled trials are needed to confirm their clinical efficacy, determine optimal dosing, and assess their potential role as adjunctive therapy in chronic disease prevention and management.

## Figures and Tables

**Table 1:** Summary of studies with Anthocyanin-EBB.

Reference	Study Type	Sample	Intervention	Results
Qin et al. (Am J Clin Nutr. 2009) [55]	Double-blind, placebo-controlled clinical study	N = 120 Patients with dyslipidemia aged 40–65 years without CVD	Anthocyanin-EBB 4 capsules/day (320 mg/day) or placebo for 12 weeks	HDLc: +13.7%. LDLc: −13.6%. Cholesterol efflux: +20%. Reduced concentration and activity of CETP.
Zhu et al. (Clin Chem. 2011) [56]	Double-blind, placebo-controlled clinical study series	N = 150 + 12 + 6 Patients with hypercholesterolemia	Anthocyanin-EBB 4 capsules/day (320 mg/day) or placebo for 12 weeks	Increased FMD from 1 h after Anthocyanin-EBB consumption and up to 28.4% after 12 weeks. cGMP: +12.6%. HDLc: +12.8%. LDLc: −10%. sVCAM1: −11.6%.
Zhu et al. (Nutr Metab Cardiovasc Dis. 2013) [57]	Double-blind, placebo-controlled clinical study	N = 150 patients with moderate hypercholesterolemia, age 40–65 years	Anthocyanin-EBB 4 capsules/day (320 mg/day) or placebo for 24 weeks	Decrease in inflammatory parameters (hsCRP: −21.6%; sVCAM-1: −12.3%; plasma IL-1β: −12.8%). LDLc: −10.4%. HDLc: +14.0%. Decrease in IL-6, IL-1β and VCAM-1 (in vitro).
Hassellund et al. (J Hum Hypertens. 2013) [58]	Double-blind, placebo-controlled crossover clinical study	N = 31, men between 35 and 51 years, with blood pressure >140/90 mmHg without antihypertensive treatment	Anthocyanin-EBB 8 capsules/day (640 mg/day) or placebo for 4 weeks	HDLc increase. No changes in LDLc. Increase in von Willebrand factor.
Zhu et al. (J Clin Endocrin Metab. 2014) [59]	Double-blind, placebo-controlled clinical study	N = 122 patients with hypercholesterolemia	Anthocyanin-EBB 4 capsules/day (320 mg/day) or placebo for 24 weeks	HDLc: +11.39%. LDLc: −9.72%. HDLc-PON1 activity: +17.4%. Cholesterol efflux: +20%.
Li et al. (J Nutr. 2015) [60]	Double-blind, placebo-controlled clinical study	N = 58 Patients with type 2 diabetes, age 56–67 years	Anthocyanin-EBB 4 capsules/day (320 mg/day) or placebo for 24 weeks	LDLc: −7.9%. TG: −23.0%. ApoB-48: −16.5%. ApoC-III: −11.0%. HDLc: +19.4%. Increased antioxidant parameters. Fasting glucose: −8.5%. Insulin resistance: −13%. Adiponectin: +23.4%. B-hydroxybutyrate: +42.4%.
Yang et al. (Nutrients. 2017) [61]	Double-blind, placebo-controlled clinical study	N = 160 untreated prediabetic or early diabetic subjects, age 40–75 years	Anthocyanin-EBB 4 capsules/day (320 mg/day) or placebo for 12 weeks	HbA1c: −14%. LDLc: −0.2 mmol/L. Apo A1: −0.09 g/L. Apo B: −0.07 g/L.
Headley et al. (Evonik. 2019) [62]	Meta-analysis	N = 684	Anthocyanin-EBB 4 capsules/day (320 mg/day) or placebo for 4–24 weeks	LDLc: −12.36%. HDLc: +5.67%. ApoB: −6.40 mg/dL. ApoA1: +4.89 mg/dL.
Xu et al. (Eur J Clin Nutr. 2021) [63]	Double-blind, placebo-controlled clinical study	N = 176 patients with dyslipidemia, age 35–70 years.	Anthocyanin-EBB at 40, 80 or 320 mg/day or placebo for 12 weeks	Anthocyanin-EBB 320 mg/day increased cholesterol efflux (+35.8%), HDLc (+0.07 mmol/L), and ApoA-I (+0.07 g/L). Dose–response relationship with cholesterol efflux (P = 0.002), HDLc (P = 0.038) and ApoA-I (P = 0.023). Improved cholesterol efflux was positively correlated with increased HDLc (r = 0.215) and ApoA-I (r = 0.327).
Tian et al. (EBioMedicine. 2021) [64]	Double-blind, placebo-controlled clinical study	N = 93 patients with dyslipidemia	Anthocyanin-EBB 40, 80, 160 or 320 mg/day or placebo for 6–12 weeks	Dose-dependent effect: 80 mg reduced platelet activation by GP IIbIIIa and reduces levels of 8-iso-PGF2α. 160 mg reduced platelet activation by GP IIbIIIa, ADP and collagen and reduced levels of 8-iso- PGF2α, MDA, ROS in platelets and increased TMRM levels in platelets. 320 mg reduced platelet activation by GP IIbIIIa ADP, collagen P-selectin and reduces levels of 8-iso-PGF2α, MDA, ROS in platelets and increased total SOD and TMRM levels in platelets. It also increased the levels of HDLc and ApoA-1.
Aboonabi et al. (Free Radic Biol Med. 2020) [65]	Open-label, prospective study of two cohorts with the same intervention	N = 35, divided into two groups: healthy subjects (Control) (N = 15) and those with MS (N = 20), age 25–75 years	Anthocyanin-EBB 4 capsules/day (320 mg/day) for 4 weeks	Anthocyanin-EBB decreased fasting glucose, TG, cholesterol and LDLc in MS patients compared to control patients. PPAR-γ expression increased, with a negative correlation with glucose, cholesterol, TG and LDLc in MS patients. Decreased hs-CRP levels in MS patients. Decreased expression of NF-kB-dependent genes (TNF-α, IL-6, IL-1A, PCAM-1 and COX2) in both groups.
Zhao et al. (Clin Nutr. 2021) [66]	Double-blind, placebo-controlled clinical study	N = 169 patients with dyslipidemia	Anthocyanin-EBB 40, 80 or 320 mg/day or placebo for 12 weeks	Anthocyanin-EBB decreased the levels of plasma ceramides in a dose-dependent manner, particularly Cer 16:0 and Cer 24:0. Improved levels of plasma lipids. Increased cholesterol efflux in subjects with dyslipidemia. Plasma reductions in Cer 16:0 and Cer 24:0 correlated in a dose-dependent manner with improvements in lipid profile and cholesterol efflux.
Rodriguez-Mateos et al. (J Gerontol A Biol Sci Med Sci. 2019) [67]	Double-bind, controlled, crossover clinical study series	N = 5 and 10 Healthy volunteers	Study 1: control drinks vs. drink with 2 capsules/day (160 mg/day) of anthocyanin-EBB for 4–5 weeks Study 2: anthocyanin-EBB 1–5 capsules/day (80, 160, 240, 320 o 480 mg/day) or placebo for 5–6 weeks	160 mg of pure Anthocyanin-EBB (2 capsules) increased FMD by a similar magnitude to blueberries containing 150 mg of Anthocyanin-EBB. Dose dependent increase in FMD after Anthocyanin-EBB consumption at 2 and 6 h.
Thompson et al. (Br J Nutr. 2017) [68]	Double-blind, placebo-controlled crossover clinical study	N = 16 Sedentary population	Anthocyanin-EBB 4 capsules/day (320 mg/day) or placebo for 4 weeks	Anthocyanin-EBB reduced the formation of monocyte and platelet aggregates (−39%). Anthocyanin-EBB inhibited the expression of PECAM-1 (−14%); PAC-1 (−10%) and P-selectin (−14%). Anthocyanin-EBB reduced the platelet aggregation induced by ADP (−29%).
Thompson et al. (J Funct Foods. 2017) [69]	Double-blind, placebo-controlled crossover clinical study	N = 26 patients with overweight/obesity	Anthocyanin-EBB 4 capsules/day (320 mg/day) or placebo for 4 weeks	Reduction in PAC-1 expression. (−12%) and P-selectin expression (−9%), formation of monocyte and platelet aggregates (−29%) and PECAM-1 expression (−21%). Reduction in platelet aggregation induced by ADP (−36%), collagen (−17%) and arachidonic acid (−24%).
Aboonabi et al. (Nutr Res. 2020) [70]	Open-label, comparative study of two cohorts of patients undergoing the same intervention	N = 55, age 25–75 years, divided into two groups depending on whether they were healthy individuals or had MS	Anthocyanin-EBB 4 capsules/day (320 mg/day) for 4 weeks	Fasting blood glucose (−13.3%). TG (−24.9%). LDLc (−33.1%). hs-CRP in women (−28%). Reduced ADP-induced platelet activation configuration expressed as P-selectin (−40%).
Gaiz et al. (Altern Ther Health Med. 2022) [71]	Uncontrolled, observational study	N = 26 healthy subjects	Anthocyanin-EBB 4 capsules/day (320 mg/day) for 4 weeks	Anthocyanin-EBB reduced ADP-stimulated platelet aggregation Anthocyanin-EBB reduced mean platelet volume, mean corpuscular hemoglobin, and mean hemoglobin concentration.
Muscarà et al. (Phytother Res. 2019) [72]	In vitro study	Adipocytes	Anthocyanin-EBB	Reduced lipid accumulation and PPAR-γ. NF-kB inhibition. Reduced insulin resistance.
Yang et al. (Nutr Metab (Lond). 2020) [73]	Double-blind, placebo-controlled clinical study	N = 160 patients with prediabetes (N = 90) or newly diagnosed diabetes (n = 70) without antidiabetic treatment	Anthocyanin-EBB 4 capsules/day (320 mg/day) for 12 weeks	Anthocyanin-EBB increased adiponectin levels. Anthocyanin-EBB decreased fasting glucose levels in diabetic patients, but not in prediabetic patients.
Yang et al. (Diabetes Metab Syndr Obes. 2020) [74]	Double-blind, placebo-controlled clinical study	N = 121 patients with elevated fasting glucose levels	Anthocyanin-EBB 4 capsules/day (320 mg/day) for 12 weeks	Anthocyanin-EBB increased serum levels of IGFBP-4 fragments. Anthocyanin-EBB reduced fasting glucose levels and decreased C-peptide levels after a three-hour oral glucose tolerance test.
Yang et al. (Eur J Nutr. 2021) [75]	Double-blind, placebo-controlled clinical study	N = 160 patients with prediabetes or newly diagnosed diabetes without antidiabetic treatment (40–75 years)	Anthocyanin-EBB 4 capsules/day (320 mg/day) for 12 weeks	Anthocyanin-EBB increased adipsin levels. Anthocyanin-EBB reduced visfatin levels. Anthocyanin-EBB improved HbA1c, apo-A1 and apo-B levels.
Cimino et al. (Genes Nutr. 2013) [76]	Ex vivo experimental study	Blood from healthy subjects supplemented with anthocyanins incubated in endothelial cell cultures	Anthocyanin-EBB 2 capsules/day (160 mg/day) in a single dose	Anthocyanin-EBB activated the transcription factor Nrf2 and the cytoprotective genes regulated by Nrf2.
Zhang et al. (Redox Biol. 2020) [77]	Double-blind, placebo-controlled clinical study	N = 169 patients with dyslipidemia	Anthocyanin-EBB at 40, 80 or 320 mg/day or placebo for 12 weeks	320 mg/day improved T-SOD in 6 weeks. 40 mg/day slightly reduced IL-6, TNF-α, and 8-iso-PGF2α at 12 weeks. 80 mg/day) significantly reduced serum IL-6 (−20%), TNF-α (−11%) and 8-iso- PGF2α in urine (−27%). 320 mg/day of anthocyanin-EBB reduced serum IL-6 (−40%), TNF-α (−21%), MDA (−20%) and 8-iso-PGF2α (−37%) and 8-OHdG in urine (−36%) more than with 80 mg/day and 40 mg/day of anthocyanin-EBB.
Aboonabi et al. (Chem Biol Interact. 2020) [78]	In vitro study	Human diabetic endothelial cells	Anthocyanin-EBB	Anthocyanin-EBB decreased cytotoxicity and oxidative stress induced by hydrogen peroxide in human aortic endothelial cells and diabetic human aortic endothelial cells cell lines. Anthocyanin-EBB reduced the lipopolysaccharide-induced IL-6 in both cell lines. Anthocyanin-EBB inhibited caspase-1 activation in diabetic human aortic endothelial cells.
Guo et al. (Nutrition. 2020) [79]	Double-blind, placebo-controlled clinical study	N = 11 Healthy non-obese young adults (18–35 years)	Anthocyanin-EBB 0, 20, 40, 80, 160 or 320 mg/day or placebo for 14 days	All doses of anthocyanin-EBB were safe. Anthocyanin-EBB reduced plasma glucose levels (groups 40, 80, 160 and 320 mg). Anthocyanin-EBB reduced plasma levels of IL-10 (doses 160 and 320 mg). Anthocyanin-EBB reduced plasma levels of IL-6 (dose 40 mg). Anthocyanin-EBB reduced 8-iso-PGF2α levels (doses 80 and 160 mg). There was a strong dose-effect relationship with IL-10 (inflammatory marker) and 8-iso- PGF2α (oxidative stress marker).
Karlsen et al. (J Nutr. 2007) [80]	Double-blind, placebo-controlled clinical study + in vitro study	N = 120, healthy women and men between 40 and 74 years old	Anthocyanin-EBB 4 capsules/day (300 mg/day) or placebo for 3 weeks	IL-8: −45%. RANTES: −15%. INFα: −40%. IL-4: −60%. IL-13: −38%. Inhibition NF-kB (in vitro).
Park et al. (Food Chem Toxicol. 2007) [81]	In *vivo* study	Asthmatic mice	Anthocyanin-EBB	Dose-dependent reduction in inflammatory parameters (lipid peroxidation, enhanced pause, glycoprotein and proliferating cell nuclear antigen, various cytokines and cyclooxygenase 2) in the lungs.
Vugic et al. (J Funct Foods. 2019) [82]	Prospective open-label study	N = 35, divided into three groups: lean (N = 15), overweight (N = 10) and obese subjects (N = 10).	Anthocyanin-EBB 4 capsules/day (320 mg/day) for 4 weeks	Anthocyanin-EBB reduced plasma levels of chemokine (C-C motif) ligand 2 in the lean, overweight, and obese groups. Anthocyanin-EBB reduced IL-6 in the obese group.
Anwar et al. (Mol Med Rep. 2016) [83]	In vitro study	Human colorectal cancer cell line	Anthocyanin-EBB	Anthocyanin-EBB supplementation decreased proliferation. Anthocyanin-EBB induced apoptosis by activation of caspase-3, activated p21Waf/Cif1. Anthocyanin-EBB increased reactive oxygen species and total cell antioxidant status.
Tang et al. (J Agric Food Chem. 2015) [84]	In vivo study	Mice	Anthocyanin-EBB	Anthocyanin-EBB reduced inflammation, oxidative stress, steatosis and fibrosis. Anthocyanin-EBB promoted the activation of AMPK and PGC-1α.
Zhang et al. (Medicine. 2015) [85]	Double-blind, placebo-controlled clinical study	N = 74 patients with nonalcoholic fatty liver disease	Anthocyanin-EBB 4 capsules/day (320 mg/day) or placebo for 12 weeks	Plasma alanine aminotransferase: −19.1% Cytokeratin-18 M30 fragment: −8.8% Anthocyanin-EBB decreased in fasting glucose. Anthocyanin-EBB decreased insulin resistance.
Cristani et al. Nat Prod Res. 2016) [86]	In vivo study	Rats	Anthocyanin-EBB	Anthocyanin-EBB normalized blood activities of glutamate oxaloacetate and glutamate pyruvate transaminase. Anthocyanin-EBB prevented acetaminophen-induced plasmatic and tissular alterations in biomarkers of oxidative stress.
Parrado-Fernández et al. Biochim Biophys Acta. 2016) [87]	In vitro study	Neuronal cells	Anthocyanin-EBB	Anthocyanin-EBB prevented mitochondrial fragmentation and cytotoxicity.
Parrado-Fernández et al. (Karolinska Institutet. 2017) [88]	In vitro study	Neuronal cells	Anthocyanin-EBB	Anthocyanin-EBB prevented cytotoxicity and recovered antioxidant capacity.
Bergland et al. (Front Genet. 2019) [89]	Open-label, comparative study	N = 27 patients with cognitive impairment (n = 8) or non-obstructive stable coronary artery disease (n = 19)	Anthocyanin-EBB 4 capsules/day (320 mg/day) for 16 weeks	Significant difference between the groups for CCL-5/RANTES. Improvements were seen in memory and executive test scores.
Borda et al. (Exp Gerontol. 2024) [90]	Post hoc analysis of a phase II, double-blind, placebo-controlled randomized clinical trial [36]	N = 201 patients at high risk of dementia. Patients were divided into 2 groups according to individual inflammatory biomarker profile	Anthocyanin-EBB 4 capsules/day (320 mg/day) for 24 weeks	Group 1 (n = 89), high levels of inflammation biomarkers: anthocyanins treatment showed a statistically significant improvement in cognitive function compared to placebo at 24 weeks. Group 2 (n = 112), low levels of inflammation biomarkers: no significant differences were observed.
Borda et al. (Geroscience. 2025) [91]	Secondary analysis of [91]. Randomized, double-blind, placebo-controlled phase II trial	Sub-sample participants (n = 99), aged 60–80 years with mild cognitive impairment or cardiometabolic disorders	Anthocyanin-EBB 4 capsules/day (320 mg/day) for 24 weeks	Anthocyanin-EBB treatment was associated with significant reductions in LDLc, cardiometabolic score, CRP, IL-6, IL-1b and Inflam z-score 5.
Aarsland et al. (Am J Geriatr Psychiatry. 2023) [92]	Phase II, double-blind, placebo-controlled randomized clinical trial	N = 206, aged 60–80 years, diagnosed with either mild cognitive impairment or ≥2 cardiometabolic disorders	Anthocyanin-EBB 4 capsules/day (320 mg/day) for 24 weeks	Anthocyanin-EBB supplementation was safe and well tolerated. No significant group difference was found in episodic memory at the end of the study but statistically significant difference in slopes; the anthocyanin group improved while the placebo group worsened.

Anthocyanin-EBB: anthocyanin-rich extract from bilberries and blackcurrants; AMPK: AMP-activated protein kinase; ApoA-1, apolipoprotein A-1; ApoB, apolipoprotein B; CETP: cholesteryl ester transfer protein; CVD: cardiovascular disease; FMD: flow-mediated dilation; HDLc: high-density lipoproteins cholesterol; 8-OHdG: 8-hydroxy-2′-deoxyguanosine; hsCRP: high sensitivity C-reactive protein; IGFBP-4: insulin-like growth factor binding protein-4; IL: interleukin; 8-iso-PGF2α: 8-iso-prostaglandin F2α; LDLc: low-density lipoproteins cholesterol; MDA: malondialdehyde; MS: metabolic syndrome; NO-cGMP: nitric oxide-cyclic guanosine monophosphate; PAC-1: procaspase activating compound-1; PECAM-1: platelet endothelial cell adhesion molecule-1; PGC-1α: peroxisome proliferator-activated receptor-gamma coactivator-1α; PON1: paraoxonase 1; PPAR-γ: peroxisome proliferator-activated receptor gamma; ROS: oxygen species; SOD: superoxide dismutase; RANTES: regulated upon activation, normal T cell expressed and secreted; sVCAM1: soluble vascular cell adhesion molecule-1; TG: triglycerides; TMRM: tetramethylrhodamine methyl ester; TNF-α: tumor necrosis factor-α; T-SOD: total superoxide dismutase.

## Data Availability

No new data were created or analyzed in this study.

## Supplementary Materials

The following supporting information can be downloaded at: https://www.mdpi.com/article/10.3390/jcm15052083/s1, Table S1: Summary of studies with Anthocyanin-EBB.
