# Supplementary material for: Anthocyanin-Rich Extracts from Bilberries and Blackcurrants in Human Health: A Narrative Review of Their Anti-Inflammatory and Antioxidant Effects
Source: J Clin Med. 2026 Mar 9;15(5):2083. doi: 10.3390/jcm15052083 (PMC12985782; doi:10.3390/jcm15052083)
Supplement: Supplementary file 1 [file jcm-15-02083-s001.zip › jcm-4136289-supplementary.pdf]

Supplementary Table S1. Summary of studies with Anthocyanin-EBB.

Reference	Indication	Study Type	Sample	Intervention	Results	Conclusion
**Qin, et al (Am J Clin Nutr. 2009) [55]**	Lipid profile	Double-blind, placebo-controlled clinical study	N=120 Patients with dyslipidemia aged 40-65 years without CVD	Anthocyanin-EBB 4 capsules/day (320 mg/day) or placebo for 12 weeks	• HDLc: +13.7%.<br>• LDLc: -13.6%.<br>• Cholesterol efflux: +20%.<br>• Reduced concentration and activity of CETP.	Anthocyanin-EBB has a beneficial effect on the lipoprotein profile (action partially due to inhibition of CETP).
**Zhu, et al (Clin Chem. 2011) [56]**	Lipid profile/ Endothelial function	Double-blind, placebo-controlled clinical study series	N = 150 + 12 + 6<br>Patients with hypercholesterolemia	Anthocyanin-EBB 4 capsules/day (320 mg/day) or placebo for 12 weeks	• Increased FMD from 1h after Anthocyanin-EBB consumption and up to 28.4% after 12 weeks.<br>• cGMP: +12.6%.<br>• HDLc: +12.8%.<br>• LDLc: -10%.<br>• sVCAM1: -11.6%.	Anthocyanin-EBB supplementation improves endothelium-dependent vasodilation in individuals with hypercholesterolemia. This effect involves activation of the NO-cGMP signaling pathway, improvements in serum lipid profile, and inflammation decrease.
**Zhu, et al (Nutr Metab Cardiovasc Dis.2013) [57]**	Lipid profile/ Inflammation	Double-blind, placebo-controlled clinical study	N = 150 patients with moderate hypercholesterolemia, age 40-65 years	Anthocyanin-EBB 4 capsules/day (320 mg/day) or placebo for 24 weeks	• Decrease in inflammatory parameters (hsCRP: -21.6%; sVCAM-1: -12.3%; plasma IL-1β: -12.8%).<br>• LDLc: -10.4%.<br>• HDLc: +14.0%.	Anthocyanin-EBB reduces the inflammatory response and improve the lipid profile in hypercholesterolemic subjects.

					• Decrease in IL-6, IL-1β and VCAM-1 (in vitro).	
**Hassellund et al (J Hum Hypertens. 2013) [58]**	Lipid profile/ Inflammation/ Hypertension	Double-blind, placebo-controlled crossover clinical study	N=31, men between 35-51 years, with blood pressure >140/90 mmHg without antihypertensive treatment	Anthocyanin-EBB 8 capsules/day (640 mg/day) or placebo for 4 weeks	• HDLc increase.<br>• No changes in LDLc.<br>• Increase of von Willebrand factor.	Anthocyanin-EBB increases HDLc in non-dyslipidemic prehypertensive subjects.
**Zhu, et al (J Clin Endocrin Metab. 2014) [59]**	Lipid profile	Double-blind, placebo-controlled clinical study	N=122 patients with hypercholesterolemia	Anthocyanin-EBB 4 capsules/day (320 mg/day) or placebo for 24 weeks	• HDLc: +11.39%.<br>• LDLc: -9.72%.<br>• HDLc-PON1 activity: +17.4%.<br>• Cholesterol efflux: +20%.	Anthocyanin-EBB improves the activity of HDLc-associated PON1 protein, promote antioxidant effects on HDLc and promote cholesterol efflux capacity in subjects with hypercholesterolemia.
**Li, et al (J Nutr. 2015) [60]**	Lipid profile/ Diabetes/Antioxidant	Double-blind, placebo-controlled clinical study	N=58<br>Patients with type 2 diabetes, age 56-67 years	Anthocyanin-EBB 4 capsules/day (320 mg/day) or placebo for 24 weeks	• LDLc: -7.9%.<br>• TG: -23.0%.<br>• ApoB-48: -16.5%.<br>• ApoC-III: -11.0%.<br>• HDLc: +19.4%.<br>• Increased antioxidant parameters.<br>• Fasting glucose: -8.5%.	Anthocyanin-EBB exerts beneficial metabolic effects in subjects with type 2 diabetes by improving dyslipidemia, increasing antioxidant capacity, and preventing insulin resistance.

					• Insulin resistance: -13%.<br>• Adiponectin: +23.4%.<br>• B-hydroxybutyrate: +42.4%.	
**Yang et al (Nutrients. 2017) [61]**	Lipid profile/Diabetes	Double-blind, placebo-controlled clinical study	N=160 untreated prediabetic or early diabetic subjects, age 40-75 years	Anthocyanin-EBB 4 capsules/day (320 mg/day) or placebo for 12 weeks	• HbA1c: -14%.<br>• LDLc: -0.2 mmol/L.<br>• Apo A1: -0.09 g/L.<br>• Apo B: -0.07 g/L.	Anthocyanin-EBB improves glycemic control and lipid profile.
**Headley, et al (Evonik. 2019) [62]**	Lipid profile/Cardiovascular	Meta-analysis	N=684	Anthocyanin-EBB 4 capsules/day (320 mg/day) or placebo for 4-24 weeks	• LDLc: -12.36%.<br>• HDLc: +5.67%.<br>• ApoB: -6.40mg/dL.<br>• ApoA1: +4.89mg/dL.	Anthocyanin-EBB has a positive and constant impact on the lipid profile of those subjects at higher risk of cardiovascular disease.
**Xu, et al (Eur J Clin Nutr. 2021) [63]**	Lipid profile	Double-blind, placebo-controlled clinical study	N=176 patients with dyslipidemia, age 35-70 years.	Anthocyanin-EBB at 40, 80 or 320 mg/day or placebo for 12 weeks	• Anthocyanin-EBB 320 mg/day increased cholesterol efflux (+35.8%), HDLc (+0.07 mmol/L), and ApoA-I (+0.07 g/L).<br>• Dose-response relationship with cholesterol efflux (P = 0.002), HDLc (P = 0.038) and ApoA-I (P = 0.023).<br>• Improved cholesterol efflux was positively correlated with	Anthocyanin-EBB supplementation doses of 80 to 320 mg/day may improve serum HDLc levels and HDLc-induced cholesterol efflux capacity. There was a dose-dependent effect.

					increased HDLc (r = 0.215) and ApoA-I (r = 0.327).	
**Tian, et al (EBioMedicine. 2021) [64]**	Lipid profile/Atherogenesis/Cardiovascular /Antioxidant	Double-blind, placebo-controlled clinical study	N=93 patients with dyslipidemia	Anthocyanin-EBB 40, 80, 160 or 320 mg/day or placebo for 6-12 weeks	• Dose-dependent effect:<br>• 80 mg reduced platelet activation by GP IIbIIIa and reduces levels of 8-iso-PGF2α.<br>• 160 mg reduced platelet activation by GP IIbIIIa, ADP and collagen and reduced levels of 8-iso- PGF2α, MDA, ROS in platelets and increased TMRM levels in platelets.<br>• 320mg reduced platelet activation by GP IIbIIIa ADP, collagen P-selectin and reduces levels of 8-iso-PGF2α, MDA, ROS in platelets and increased total SOD and TMRM levels in platelets. It also increased the levels of HDLc and ApoA-1.	Anthocyanin-EBB ≥80 mg for 12 weeks reduces platelet activation and aggregation and oxidative stress in a dose-dependent manner in subjects with dyslipidemia.
**Aboonabi, et al (Free Radic Biol Med. 2020) [65]**	Lipid profile/MS/Inflammation	Open-label, prospective study of two cohorts with the same intervention	N=35, divided into two groups: healthy subjects (Control) (N=15) and those	Anthocyanin-EBB 4 capsules/day (320 mg/day) for 4 weeks	• Anthocyanin-EBB decreased fasting glucose, TG, cholesterol and LDLc in MS patients compared to control patients.	Anthocyanin-EBB improves selected features of MS and related cardiovascular risk factors. These benefits may be due to the inhibition of NF-κB-dependent gene expression and activation of PPAR-γ.

			with MS (N=20), age 25-75 years		• PPAR-γ expression increased, with a negative correlation with glucose, cholesterol, TG and LDLc in MS patients.<br>• Decreased hs-CRP levels in MS patients.<br>• Decreased expression of NF-kB-dependent genes (TNF-α, IL-6, IL-1A, PCAM-1 and COX2) in both groups.	
**Zhao, et al (Clin Nutr. 2021) [66]**	Lipid profile	Double-blind, placebo-controlled clinical study	N=169 patients with dyslipidemia	Anthocyanin-EBB 40, 80 or 320 mg/day or placebo for 12 weeks	• Anthocyanin-EBB decreased the levels of plasma ceramides in a dose-dependent manner, particularly Cer 16:0 and Cer 24:0.<br>• Improved levels of plasma lipids.<br>• Increased cholesterol efflux in subjects with dyslipidemia.<br>• Plasma reductions of Cer 16:0 and Cer 24:0 correlated in a dose-dependent manner with improvements in lipid profile and cholesterol efflux.	Anthocyanin-EBB may help improve metabolic and cardiovascular health in part by modulating circulating levels of ceramides.

**Rodriguez-Mateos, et al (J Gerontol A Biol Sci Med Sci. 2019) [67]**	Endothelial function	Double-bind, controlled, crossover clinical study series	N=5 and 10<br>Healthy volunteers	• Study 1: control drinks vs drink with 2 capsules/day (160 mg/day) of anthocyanin-EBB for 4-5 weeks<br>• Study 2: anthocyanin-EBB 1-5 capsules/day (80, 160, 240, 320 o 480 mg/day) or placebo for 5-6 weeks	• 160 mg of pure Anthocyanin-EBB (2 capsules) increased FMD by a similar magnitude to blueberries containing 150 mg of Anthocyanin-EBB.<br>• Dose dependent increase in FMD after Anthocyanin-EBB consumption at 2 and 6 hours.	Study 1: Anthocyanin-EBB is the major contributors to improved endothelial function after blueberry consumption.<br>Study 2: Anthocyanin-EBB produces a dose-dependent increase in FMD.
**Thompson, et al (Br J Nutr. 2017) [68]**	Atherogenesis	Double-blind, placebo-controlled crossover clinical study	N=16<br>Sedentary population	Anthocyanin-EBB 4 capsules/day (320 mg/day) or placebo for 4 weeks	• Anthocyanin-EBB reduced the formation of monocyte and platelet aggregates (-39%).<br>• Anthocyanin-EBB inhibited the expression of PECAM-1 (-14%);	Anthocyanin-EBB has the potential to alleviate biomarkers of atherogenesis, platelet aggregation and hyperactivation in the sedentary population.

					PAC-1 (-10%) and P-selectin (-14%).<br>• Anthocyanin-EBB reduced the platelet aggregation induced by ADP (-29%).	
**Thompson, et al (J Funct Foods. 2017) [69]**	Atherogenesis/ Overweight	Double-blind, placebo-controlled crossover clinical study	N = 26 patients with overweight/obesity	Anthocyanin-EBB 4 capsules/day (320 mg/day) or placebo for 4 weeks	• Reduction of PAC-1 expression.<br>• (-12%) and P-selectin expression (-9%), formation of monocyte and platelet aggregates (-29%) and PECAM-1 expression (-21%).<br>• Reduction in platelet aggregation induced by ADP (-36%), collagen (-17%) and arachidonic acid (-24%).	Anthocyanin-EBB has the potential to reduce the risk of thrombosis in the overweight/obese population by targeting specific pathways of platelet activation/aggregation and leukocyte migration associated with endothelial dysfunction.
**Aboonabi, et al (Nutr Res. 2020) [70]**	Atherogenesis/ Cardiovascular	Open-label, comparative study of two cohorts of patients undergoing the same intervention	N=55, age 25-75 years, divided into two groups depending on whether they were healthy individuals or had MS	Anthocyanin-EBB 4 capsules/day (320 mg/day) for 4 weeks	• Fasting blood glucose (-13.3%).<br>• TG (-24.9%).<br>• LDLc (-33.1%).<br>• hs-CRP in women (-28%).<br>• Reduced ADP-induced platelet activation configuration expressed as P-selectin (-40%).	Anthocyanin-EBB exerts antiatherogenic effects by improving cardiometabolic risk factors and reducing thrombogenicity in the population with MS.

**Gaiz, et al (Altern Ther Health Med. 2022) [71]**	Atherogenesis	Uncontrolled, observational study	N=26 healthy subjects	Anthocyanin-EBB 4 capsules/day (320 mg/day) for 4 weeks	• Anthocyanin-EBB reduced ADP-stimulated platelet aggregation<br>• Anthocyanin-EBB reduced mean platelet volume, mean corpuscular hemoglobin, and mean hemoglobin concentration.	Anthocyanin-EBB inhibits platelet activity, platelet aggregation, and mean platelet volume. These results suggest that anthocyanins have a positive impact on the attenuation of platelet activity, which could minimize thrombotic risk.
**Muscarà, et al (Phytother Res. 2019) [72]**	Diabetes/Inflammation	*In vitro* study	Adipocytes	Anthocyanin-EBB	• Reduced lipid accumulation and PPAR-γ.<br>• NF-kB inhibition.<br>• Reduced insulin resistance.	Anthocyanin-EBB demonstrates potential preventive effects against obesity and related comorbidities, due to their protective effects against inflammation/resistance insulin in adipocytes.
**Yang, et al (Nutr Metab (Lond). 2020) [73]**	Diabetes	Double-blind, placebo-controlled clinical study	N=160 patients with prediabetes (n=90) or newly diagnosed diabetes (n=70) without antidiabetic treatment	Anthocyanin-EBB 4 capsules/day (320 mg/day) for 12 weeks	• Anthocyanin-EBB increased adiponectin levels.<br>• Anthocyanin-EBB decreased fasting glucose levels in diabetic patients, but not in prediabetic patients.	Anthocyanin-EBB supplementation for 12 weeks improves fasting serum adiponectin and glucose levels in newly diagnosed diabetic patients, but not in prediabetic patients.
**Yang, et al (Diabetes Metab Syndr Obes. 2020) [74]**	Diabetes	Double-blind, placebo-controlled clinical study	N=121 patients with elevated fasting glucose levels	Anthocyanin-EBB 4 capsules/day (320 mg/day) for 12 weeks	• Anthocyanin-EBB increased serum levels of IGFBP-4 fragments.<br>• Anthocyanin-EBB reduced fasting glucose levels and decreased C-peptide levels after	Anthocyanin-EBB improves serum levels of IGFBP-4 fragments and reduce fasting glucose levels and C-peptide after oral glucose tolerance test.

					a three-hour oral glucose tolerance test.	
**Yang, et al (Eur J Nutr. 2021) [75]**	Diabetes	Double-blind, placebo-controlled clinical study	N=160 patients with prediabetes or newly diagnosed diabetes without antidiabetic treatment (40-75 years)	Anthocyanin-EBB 4 capsules/day (320 mg/day) for 12 weeks	• Anthocyanin-EBB increased adipsin levels.<br>• Anthocyanin-EBB reduced visfatin levels.<br>• Anthocyanin-EBB improved HbA1c, apo-A1 and apo-B levels.	Improvement in serum adipsin may slow the progression of diabetes.<br>The improvements in visfatin, apo A1, and apo B suggest antiatherogenic and cardioprotective properties of Anthocyanin-EBB.
**Cimino, et al (Genes Nutr. 2013) [76]**	Antioxidant	*Ex vivo* experimental study	Blood from healthy subjects supplemented with anthocyanins incubated in endothelial cell cultures	Anthocyanin-EBB 2 capsules/day (160 mg/day) in a single dose	• Anthocyanin-EBB activated the transcription factor Nrf2 and the cytoprotective genes regulated by Nrf2.	Anthocyanin-EBB and their metabolites can protect endothelial cells, not because of their antioxidant properties, but modulation of cell signaling by inducing the activation of the Nrf2/ARE pathway.
**Zhang, et al (Redox Biol. 2020) [77]**	Antioxidant/Inflammation	Double-blind, placebo-controlled clinical study	N=169 patients with dyslipidemia	Anthocyanin-EBB at 40, 80<br>or 320 mg/day or placebo for 12 weeks	• 320 mg/day improved T-SOD in 6 weeks.<br>• 40 mg/day slightly reduced IL-6, TNF-α, and 8-iso-PGF2α at 12 weeks.<br>• 80 mg/day) significantly reduced serum IL-6 (−20%),	Anthocyanin-EBB supplementation for 12 weeks positively improves antioxidant and anti-inflammatory capacity in a dose-response manner in individuals with dyslipidemia. 80 mg/day could reach the threshold level to reach beneficial effects among subjects with dyslipidemia. The duration of anthocyanin supplementation plays an

					TNF-α (−11%) and 8-iso- PGF2α in urine (−27%).<br>• 320 mg/day of anthocyanin-EBB reduced serum IL-6 (−40%), TNF-α (−21%), MDA (−20%) and 8-iso-PGF2α (−37%) and 8-OHdG in urine (−36%) more than with 80 mg/day and 40 mg/day of anthocyanin-EBB.	important role in the prevention of metabolic diseases.
**Aboonabi, et al (Chem Biol Interact. 2020) [78]**	Antioxidant/Inflammation/Diabetes	*In vitro* study	Human diabetic endothelial cells	Anthocyanin-EBB	• Anthocyanin-EBB decreased cytotoxicity and oxidative stress induced by hydrogen peroxide in human aortic endothelial cells and diabetic human aortic endothelial cells cell lines.<br>• Anthocyanin-EBB reduced the lipopolysaccharide-induced IL-6 in both cell lines.<br>• Anthocyanin-EBB inhibited caspase-1 activation in diabetic human aortic endothelial cells.	Anthocyanin-EBB reduces oxidative stress and inflammation through inhibition of the NF-ƙB signaling pathway, which contributes to mitigating diabetes-induced upregulation of NF-ƙB.
**Guo, et al (Nutrition. 2020) [79]**	Antioxidant/Inflammation	Double-blind, placebo-controlled clinical study	N=11 Healthy non-obese young adults (18-35 years)	Anthocyanin-EBB 0, 20, 40, 80, 160 or 320 mg/day or	• All doses of anthocyanin-EBB were safe.	Providing healthy men and women with dietary Anthocyanin-EBB in doses above 80 mg/d improves some markers of oxidative stress and inflammation.

				placebo for 14 days	• Anthocyanin-EBB reduced plasma glucose levels (groups 40, 80, 160 and 320 mg).<br>• Anthocyanin-EBB reduced plasma levels of IL-10 (doses 160 and 320 mg).<br>• Anthocyanin-EBB reduced plasma levels of IL-6 (dose 40 mg).<br>• Anthocyanin-EBB reduced 8-iso-PGF2α levels (doses 80 and 160 mg).<br>• There was a strong dose-effect relationship with IL-10 (inflammatory marker) and 8-iso- PGF2α (oxidative stress marker).	
**Karlsen, et al (J Nutr. 2007) [80]**	Inflammation	Double-blind, placebo-controlled clinical study + *in vitro* study	N=120, healthy women and men between 40-74 years old	Anthocyanin-EBB 4 capsules/day (300 mg/day) or placebo for 3 weeks	• IL-8: -45%.<br>• RANTES: -15%.<br>• INFα: -40%.<br>• IL-4: -60%.<br>• IL-13: -38%.<br>• Inhibition NF-kB (in vitro).	Anthocyanin-EBB exerts an anti-inflammatory effect by reducing levels of NF-kB-dependent pro-inflammatory cytokines.

**Park et al (Food Chem Toxicol. 2007) [81]**	Inflammation/Respiratory	In *vivo* study	Asthmatic mice	Anthocyanin-EBB	• Dose-dependent reduction of inflammatory parameters (lipid peroxidation, enhanced pause, glycoprotein and proliferating cell nuclear antigen, various cytokines and cyclooxygenase 2) in the lungs.	Anthocyanin-EBB may attenuate the development of asthma by downregulating Th2 cytokines, pro-inflammatory cytokines, and cyclooxygenase 2.
**Vugic, et al (J Funct Foods. 2019) [82]**	Inflammation/ Overweight	Prospective open-label study	N=35, divided into three groups: lean (N=15), overweight (N=10) and obese subjects (N=10).	Anthocyanin-EBB 4 capsules/day (320 mg/day) for 4 weeks	• Anthocyanin-EBB reduced plasma levels of chemokine (C-C motif) ligand 2 in the lean, overweight, and obese groups.<br>• Anthocyanin-EBB reduced IL-6 in the obese group.	Anthocyanin-EBB reduces inflammatory markers and suggest their use as an adjunctive therapy to reduce chronic inflammation in obese and overweight people.
**Anwar, et al (Mol Med Rep. 2016) [83]**	Oncology	*In vitro* study	Human colorectal cancer cell line	Anthocyanin-EBB	• Anthocyanin-EBB supplementation decreased proliferation.<br>• Anthocyanin-EBB induced apoptosis by activation of caspase-3, activated p21Waf / Cif1.<br>• Anthocyanin-EBB increased reactive oxygen species and total cell antioxidant status.	Anthocyanin-EBB inhibits proliferation of Caco-2 human colorectal cancer cells by promoting ROS accumulation, inducing caspase-3 activation, and upregulating the expression of p21Waf/Cif1

**Tang, et al (J Agric Food Chem. 2015) [84]**	Liver health	*In vivo* study	Mice	Anthocyanin-EBB	• Anthocyanin-EBB reduced inflammation, oxidative stress, steatosis and fibrosis.<br>• Anthocyanin-EBB promoted the activation of AMPK and PGC-1α.	Anthocyanin-EBB exerts protective effects against non-alcoholic hepatic steatosis and mitochondrial defects in response to a methionine-deficient diet, with a mechanism that may imply AMPK/PGC-1α signaling pathways.
**Zhang, et al (Medicine. 2015) [85]**	Liver health	Double-blind, placebo-controlled clinical study	N=74 patients with nonalcoholic fatty liver disease	Anthocyanin-EBB 4 capsules/day (320 mg/day) or placebo for 12 weeks	• Plasma alanine aminotransferase: -19.1%<br>• Cytokeratin-18 M30 fragment: -8.8%<br>• Anthocyanin-EBB decreased in fasting glucose.<br>• Anthocyanin-EBB decreased insulin resistance.	Anthocyanin-EBB improves insulin resistance, liver damage indicators, and clinical outcome in patients with nonalcoholic fatty liver disease.
**Cristani, et al. Nat Prod Res. 2016) [86]**	Liver health	*In vivo* study	Rats	Anthocyanin-EBB	• Anthocyanin-EBB normalized blood activities of glutamate oxaloacetate and glutamate pyruvate transaminase.<br>• Anthocyanin-EBB prevented acetaminophen-induced plasmatic and tissular alterations in biomarkers of oxidative stress.	The observed effect is probably due to various bioproperties of the components of the anthocyanin-EBB.

**Parrado-Fernández, et al. Biochim Biophys Acta. 2016) [87]**	Cognitive health	*In vitro* study	Neuronal cells	Anthocyanin-EBB	• Anthocyanin-EBB prevented mitochondrial fragmentation and cytotoxicity.	Anthocyanin-EBB, in addition to their antioxidant capacity, improves mitochondrial dysfunction generated by overproduction of amyloid-β peptide or by chemical inhibition of mitochondrial complex I by stabilizing the processes of fusion and fission proteins.
**Parrado-Fernández, et al (Karolinska Institutet. 2017) [88]**	Cognitive health	*In vitro* study	Neuronal cells	Anthocyanin-EBB	• Anthocyanin-EBB prevented cytotoxicity and recovered antioxidant capacity.	Anthocyanin-EBB could slow cognitive decline in middle-aged and older people with pre- Alzheimer disease dementia or mild Alzheimer disease dementia and in the coronary artery disease.
**Bergland, et al (Front Genet. 2019) [89]**	Cognitive health/Lipid profile/Glucose /Inflammation	Open-label, comparative study	N=27 patients with cognitive impairment (n=8) or non-obstructive stable coronary artery disease<br><br>(n=19)	Anthocyanin-EBB 4 capsules/day (320 mg/day) for 16 weeks	• Significant difference between the groups for CCL-5/RANTES.<br>• Improvements were seen in memory and executive test scores.	Inconclusive results
**Borda, et al (Exp Gerontol. 2024) [90]**	Inflammation/Cognitive health	Post-hoc analysis of a phase II, double-blind, placebo-controlled randomized clinical trial [36]	N=201 patients at high risk of dementia.<br><br>Patients were divided into 2 groups according to individual	Anthocyanin-EBB 4 capsules/day (320 mg/day) for 24 weeks	• Group 1 (n = 89), high levels of inflammation biomarkers: anthocyanins treatment showed a statistically significant improvement in cognitive function compared to placebo at 24 weeks.	Anthocyanin-EBB improves cognitive function in those patients who have elevated levels of inflammatory markers compared to placebo at 24 weeks.

			inflammatory biomarker profile		• Group 2 (n = 112), low levels of inflammation biomarkers: no significant differences were observed.	
**Borda, et al (Geroscience. 2025) [91]**	Inflammation/Cognitive health	Secondary analysis of [91]. Randomized, double-blind, placebo-controlled phase II trial	Sub-sample participants (n = 99), aged 60-80 years with mild cognitive impairment or cardiometabolic disorders	Anthocyanin-EBB 4 capsules/day (320 mg/day) for 24 weeks	• Anthocyanin-EBB treatment was associated with significant reductions in LDLc, cardiometabolic score, CRP, IL - 6, IL - 1b and Inflam z-score 5.	Anthocyanin-EBB supplementation reduces CRP and cardiovascular disease biomarkers in individuals at risk of dementia.
**Aarsland, et al (Am J Geriatr Psychiatry. 2023) [92]**	Cognitive health	Phase II, double-blind, placebo-controlled randomized clinical trial	N= 206, aged 60-80 years, diagnosed with either mild cognitive impairment or ≥2 cardiometabolic disorders	Anthocyanin-EBB 4 capsules/day (320 mg/day) for 24 weeks	• Anthocyanin-EBB supplementation was safe and well tolerated. • No significant group difference was found in episodic memory at the end of the study but statistically significant difference in slopes; the anthocyanin group improved while the placebo group worsened.	There was a trend towards improvement in episodic memory with anthocyanin-EBB.

Anthocyanin-EBB: Anthocyanin-Rich Extracts from Bilberries and Blackcurrants; AMPK: AMP-activated protein kinase; ApoA-1, apolipoprotein A-1; ApoB, apolipoprotein B; CETP: cholesteryl ester transfer protein; CVD: cardiovascular disease; FMD: flow-mediated dilation; HDLc: high-density lipoproteins cholesterol; 8-OHdG: 8-hydroxy-2'-deoxyguanosine; hsCRP: high sensitivity C-reactive protein; IGFBP-4: insulin-like growth factor binding protein-4; IL: interleukin; 8-iso-PGF2α: 8-iso-prostaglandin F2α; LDLc: low-density lipoproteins cholesterol; MDA: malondialdehyde; MS: metabolic syndrome; NO-cGMP: nitric oxide- cyclic guanosine monophosphate; PAC-1: procaspase activating compound-1; PECAM-1: platelet endothelial cell adhesion molecule-1; PGC-1α: peroxisome proliferator-activated receptor-gamma coactivator-1α; PON1: Paraoxonase 1; PPAR-γ: Peroxisome Proliferator-Activated Receptor gamma; ROS: oxygen species; SOD: superoxide dismutase; RANTES: regulated upon activation, normal T

cell expressed and secreted; sVCAM1: soluble Vascular Cell Adhesion Molecule-1; TG: triglycerides; TMRM: tetramethylrhodamine methyl ester; TNF-α: tumor necrosis factor-α; T-SOD: total superoxide dismutase.
